# The effects of a life goal-setting technique in a preventive care program for frail community-dwelling older people: a cluster nonrandomized controlled trial

**DOI:** 10.1186/s12877-016-0277-3

**Published:** 2016-05-12

**Authors:** Yoshimi Yuri, Shinichi Takabatake, Tomoko Nishikawa, Mari Oka, Taro Fujiwara

**Affiliations:** Department of Rehabilitation Sciences, Faculty of Allied Health Sciences, Kansai University of Welfare Sciences, Osaka, Japan; Graduate School of Comprehensive Rehabilitation, Osaka Prefecture University, Osaka, Japan; Elderly Care Office, Izumi, Osaka Japan; Izumi Rehabilitation Home-Visit Nursing Care Station, Osaka, Japan

**Keywords:** Living in the community, Frail older people, Cluster nonrandomized controlled trial, Goal-setting

## Abstract

**Background:**

Frailty among older people is associated with an increased risk of needing care. There have been many reports on preventive care programs for frail older people, but few have shown positive effects on disability prevention. Physical exercise programs for frail older people affect elements such as physical fitness and balance, but are less effective for disability outcomes and are not followed up in the longer term. We developed a life goal-setting technique (LGST). Our objective was to determine the effect of a LGST plus standard preventive care program for community-dwelling frail older people.

**Methods:**

We used a cluster nonrandomized controlled trial with seven intervention and nine matched control groups, with baseline assessment and follow-up at 3, 6, and 9 months. Participants were 176 frail older people, aged 65 years or over, living in the community in Izumi, Osaka, Japan. All participants attended regular 120 min preventive care exercise classes each week, over 3 months. They also received oral care and nutrition education. The intervention groups alone received life goal-setting support. We assessed outcomes longitudinally, comparing pre-intervention with follow-up. The primary outcome measure was health improvement according to the Japanese Ministry of Health, Labour and Welfare’s “Kihon Checklist” for assessment of frailty and quality of life (QOL), analyzed with a two-way ANOVA and post-test comparison. Secondary outcomes included physical functions and assessment of life goals.

**Results:**

The improvement on the Kihon Checklist for the intervention group was approximately 60 % from baseline to 9-months follow-up; the control group improved by approximately 40 %. The difference between groups was significant at 3-month (*p* = 0.043) and 6-month (*p* = 0.015) follow-ups but not at 9-month (*p* = 0.098) follow-up. Analysis of QOL yielded a significant time × group interaction effect (*p* = 0.022). The effect was significant at 3 months in the intervention group, but at no time in the control group.

**Conclusion:**

A 3-month exercise program helped to decrease frailty and improve QOL in frail older people, and the addition of LGST increased its effectiveness. The LGST is a feasible and promising intervention for reducing risk of needing care.

**Trial registration:**

UMIN000021485. Registered 15 March 2016.

## Background

Frailty among older people is associated with an increased risk of adverse health outcomes such as acute and chronic diseases, disability and mortality [1–3]. Frailty has also been associated with a significant impairment in quality of life (QOL) [4]. To maintain independent living, people need to have sustained function, including daily life functions, cognitive function, emotion and sociality [5]. Many preventive care programs for frail older people have been reported. Reviews have shown that physical exercise programs for frail older people mostly affect issues such as physical fitness and balance, but are less effective on disability outcomes and are not followed up in the longer term [5–8]. It is important to develop an effective intervention to prevent disability and improve participation for frail older people living in the community.

A literature review by Daniels et al. [6] showed that relatively long-lasting and high-intensity multicomponent exercise programs have a positive effect on activities of daily living (ADL), instrumental activities of daily living (IADL) and disability for frail older people living in the community. It is suggested [6] that future community care interventions for frail older people be directed towards tailor-made, multidisciplinary and multifactorial interventions, with individualized assessment and long-term follow-up.

We researched methods that had long-lasting effects of raising motivation. Clark et al. [9, 10] reported some of the long- term effects of group education and counselling on independent living in older people. The study used a method whereby older people reflect on their own life activity and set life goals that fit their individual values. Locke et al. [11, 12] reported long-lasting improvements in ADL, IADL and QOL as a result of goal-setting; these were documented with goal attainment scaling methods [13, 14].

We applied the occupational evaluation technique and developed an original method, which we call the life goal-setting technique (LGST). Our objective was to determine the effect of a LGST plus standard preventive care program for community-dwelling frail older people in Japan.

## Method

### Setting and participants

**Fig. 1:**
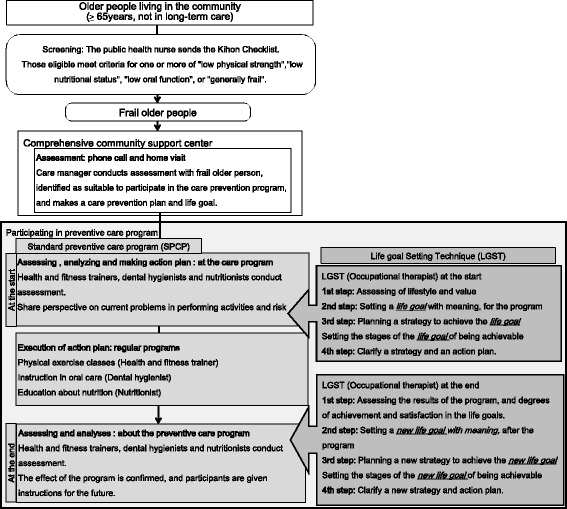
Japanese care prevention system and LGST protocol

The study was conducted in accordance with principles set out by the Japanese Ministry of Health, Labour and Welfare (MHLW) [15] (see Fig. 1). The Japanese MHLW started the Care Prevention Programs in 2006 to prevent frailty and disability of older persons, and introduced the “Kihon Checklist” (KCL) to identify frail older adults [15–20]. Those eligible were older people aged 65 or over living in the community in Izumi, Osaka, Japan, who were assessed as frail using the MHLW’s “Kihon Checklist” [19]. They were invited to join a care prevention program between October 2009 and March 2011. And they decided to participate in a care prevention program for own intention. Exclusion criteria were: 1) receiving certification for long-term care; 2) having participated in a preventive care program within past 2 years; 3) missing more than five classes out of 12 (an overall attendance rate less than 66.6 %); 4) absent on the day of life goal-setting; and 5) not returning the postal questionnaire.

The screening was carried out by a public health nurse, who sent the Kihon Checklist to older people living in the community who were ineligible to receive certification for long-term care [19]. The 25-item Kihon Checklist is the official self-administered questionnaire that comprises seven domains: activities of daily living, physical strength, nutritional status, oral function, houseboundness, cognitive function and depression risk [15, 19]. The frailty criteria were determined by the MHLW, and established by algorithm for each subject. According to Japanese frailty criteria, eligibility for this study was defined as having one or more of the following: “low physical strength,” “low nutritional status,” “low oral function,” or “generally frail” status [15, 20]. It is showing “low physical strength” if they score three or more negative responses out of five questions. “Low nutritional status” is assessed by two questions, with negative answers to both indicating lower status, and “low oral function” is defined as two or more negative responses out of three questions. And who endorse at least 10 ‘frail’ negative answers out of 1–20 questions are categorized as “generally frail” [15, 20]. All items and criteria used for screening the frail elderly are useful for predicting the risk of incidence of long-term care insurance certification during a one-year period [17].

A case manager visited each participant in their home to make a plan for preventive care management and to discuss life goals. Case managers also assessed participants’ environment and their ability to perform ADL and IADL, and took a medical history.

The programs were conducted in three places in Izumi. The standard preventive care program (SPCP) included physical exercise classes, oral care and nutrition education. In brief, the program involved learning about the training contents and actually experiencing strategies in a group setting to improve the individuals’ weak areas. Individual homework was also assigned. The fitness trainers, dental hygienists and nutritionists performed an assessment at the beginning and end of the program. Dental hygienists taught tongue exercise, salivary gland massage, deglutition, an articulation exercise, and oral hygiene 3 times each over the course of sessions. The nutritionists educated participants on nourishment (number of meals, nutritional balance, water intake, and meal contents, including supplements). Depending on the need, this information was presented once or twice over the 12 sessions. Fitness trainers implemented multi-component exercise interventions at each session. Exercise classes lasted 120 min once a week, for 12 weeks, and each class contained 10–15 participants. Each participant was assigned to either ‘LGST plus SPCP’ or ‘SPCP alone’ groups. The ‘LGST plus SPCP’ group worked with occupational therapists at the beginning and end of the program to define and then review life goals.

### Design

We conducted a nonrandomized, prospective controlled trial with seven intervention and nine matched control groups, measured at baseline and followed up 3, 6, and 9 months later. This method was used to avoid contamination bias. The public health nurse assigned participants to intervention or control classes to create an area balance. The study was quasi-experimental because assignments were not random. Intervention and control groups were well matched in average group enrollment. The instructors were asked to teach both groups using the same approach and protocols as usual. Because of the nature of the intervention, it was not possible to blind participants.

**Fig. 2:**
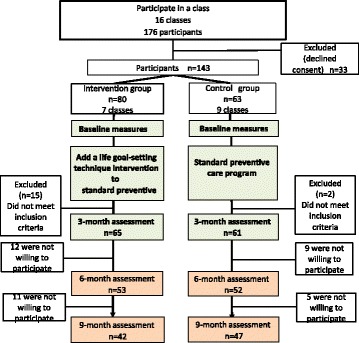
Flow diagram of the study design

The baseline period was from October 2009 to March 2011, and the study period included the first 6 months of the following year. A flow diagram of the study design is shown in Fig. 2.

### Ethical approval

Ethical approval for the study was provided by the Research Committee of the School of Comprehensive Rehabilitation at Osaka Prefecture University, and all participants gave written informed consent, including for follow-up 6 and 9 months later.

### Intervention

Based on the literature and an expert meeting, a first draft of the intervention protocol was developed by a multidisciplinary task group. This group consisted of public health nurses (PHN), occupational therapists (OT) and a researcher who served as the coordinator. The intervention puts emphasis on supporting frail older people to restore, continue or develop activities, assuming that participation in social and productive activities is protective against adverse outcomes. The preventive care program therefore assessed participants’ lifestyle and values, and helped them to set a life goal. Participants of both groups were encouraged to take regular exercise. Participants in the LGST group received life goal-setting support from the OT, as well as standard care.

Two occupational therapists worked with each group. Both were qualified at the master’s level and had 10 years of experience. Before the start of the intervention, they received training on the intervention protocol.

**Fig. 3:**
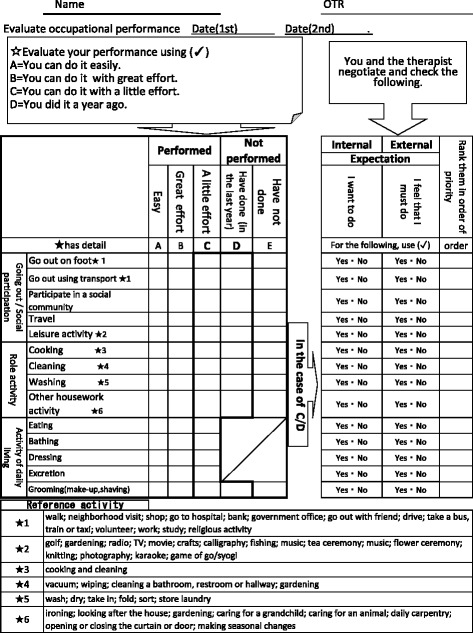
Life Goal-Setting Sheet

The life goal-setting intervention consisted of four steps (Fig. 1). At the beginning of the classroom program (step 1), the OTs assessed the participants to prepare a suitable care plan. The OT assessed each participant’s usual activities, and confirmed lifestyle and values, using a life goal-setting sheet (Fig. 3). The activities that were checked included going out, social participation, and everyday activities. The OT also identified whether the participant had done that activity in the current year, and whether the activity was easy, or required effort. To confirm a sense of the value placed on the activity, the OT identified whether there were internal or external expectations about performing the activities. Internal expectation means that the person wishes to do that activity, and external means that they feel obliged to do it.

These assessments are based on the occupational balance theory, the interest checklist [21] and the Canadian Occupational Performance Measure (COPM) [22]. After step 1, participants imagined their future, and prioritized their activities and participation to match it. They decided on their own life goal, based on lifestyle and values (step 2).

In the third step, the OT assisted the participant in planning how they would attain their goals, based on an improved understanding of motives. The OT identified existing problems in performing daily activities and risk factors for developing disability, and ensured that the plan addressed these (step 3). For the specific life goals, the OT set the stages that comprised small steps achievable during a 3-month program, then used occupational analysis and motion analysis to help identify suitable activities to attain the life goals.

**Fig. 4:**
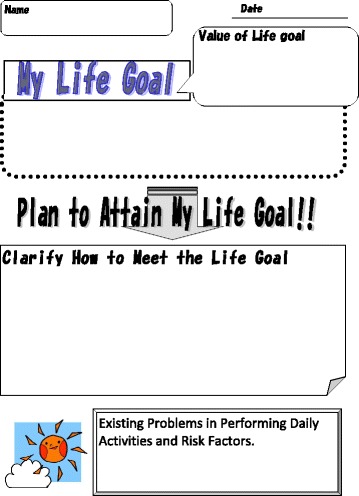
Life Goal-Setting Sheet for participants

The values recorded represented perceived status relative to self-selected goals. Both life goal content and rating were logged into the study database. This ensured that life goals were identified for activity or participation level. Specific interview questions were not provided. Instead, a framework was used to help the participants articulate their life goals. Examples of life goals were “To be able to clean my house myself”, and “To continue to go shopping on foot, reaching a supermarket within 30 min”. The next step was to clarify for participants how to attain their life goals (step 4) using the life goal-setting sheet (Fig. 4). The OT shared the life goals, a strategy and an action plan with a health and fitness trainer and other staff.

At the end of the program, the OT interviewed participants to establish the extent to which they had achieved and been satisfied with their life goals. The OT evaluated the degree of achievement of goals. Together, participants and the OT set a new life goal and clarified a new strategy and action plan, using the same methods as before (Fig. 1).

### Outcomes

#### Primary outcome measures

Primary outcomes were health improvement and QOL. The former was measured by the Kihon Checklist, which has four domains. We determined as the health improvement that were not any eligibility of frailty criteria. The latter was measured by a measure of self-rated health with a 5-point answer scale [23, 24], which captures a subjective feeling of health by asking a single question, “How would you rate your health?” with a Likert-type response scale from 1 (Poor) to 5 (Good).

#### Secondary outcome measures

We used questionnaires to assess whether participants had recognized their own life goals, and their degree of achievement and satisfaction. We asked participants, “Do you have a life goal for the program?” with the yes/no response option, followed by ‘“What is it?” (for “yes” answers). “Yes” was coded as recognition of one or more life goals, and “no” was coded as no recognition. The intervention group identified their life goals with the OT as part of the program. The control group identified life goals with the care manager, alongside their care plan. We also asked whether they had achieved their goals, and to what degree they were satisfied with them, using a scale from 1 to 10 in both domains.

Physical functions were assessed at baseline and 3 months later by health and fitness trainers who were blinded to treatment assignment. They included grip strength [25], sit and reach test [26, 27] and timed Up-and-Go test [28]. The grip strength test measured the right and left hand twice each and used the mean of the best scores from each hand. This test has been shown to be related to muscle strength. Both “sit and reach” and Timed Up-and-Go tests were measured twice to give a mean. Subjects were given rest breaks between tests.

Data on medical history and basic attributes were gathered from the records of the Izumi city government. All primary and secondary outcome measures, except physical function, were assessed at baseline and after 3 months by study staff blinded to treatment assignment. Assessments were conducted via postal questionnaires at 6 and 9 months. We used personalized questionnaires, each with a stamped return envelope, and provided non-respondents with a reminder card.

#### Sample size and power

The population sample size is based on the primary outcome measure of improvement. Based on a previous report [29], we expected to demonstrate a difference of at least 7 % in mean change in score of the frailty checklist between the intervention and control groups (equivalent to an effect size of 0.43). Based on a power of 80 % and an alpha of 0.05, we decided on a minimum sample size of *n* = 32 per group (64 in total). Based on expected drop-out rate of 50 % in the control group and 60 % in the intervention group, the required sample size was *n* = 64 for the control group and *n* = 80 for the intervention group (*n* = 144 in total).

The cluster nonrandomized design also has consequences for the sample size and power. Scores of individuals within a cluster are assumed to be correlated, in contrast to those of individuals in different clusters. A within-cluster correlation leads to a greater homogeneity of individuals within that cluster. This increases the standard error of the estimate of the treatment effect, which can result in a loss of power for detecting differences between the intervention and control group.

#### Statistical analysis

Descriptive techniques were used to describe the study groups. Baseline variables were compared to identify any differences between the intervention and control groups at the start of the study.

All analyses were conducted according to the intention-to-treat paradigm. For health improvement, we used a chi-square test to compare changes at 3, 6, and 9 months between the two groups. For QOL, group comparisons were achieved with a two-way ANOVA the three follow-up intervals. For the post hoc test, we used Dunnett’s multiple comparison tests. For the secondary outcome measures related to life goals, we calculated percentage change and used a chi-square test and Student’s *t*-test. For group comparisons on the physical functions, we used a two-way ANOVA for changes between baseline and the three follow-up intervals. For physical function improvement, we used a Student’s-*t* test.

## Results

### Recruitment feasibility, protocol adherence, and safety

Figure 2 summarizes participant study flow. We recruited 143 participants (77.6 % female) with an average age of 75.6 years (SD 5.7 years). Of these 143, 126 (88.1 %), 105 (73.4 %) and 89 (62.2 %) completed the 3-month, 6-month and 9-month assessment, respectively. 42 (52.5 %) of the intervention group and 47 (74.6 %) of the control group completed both baseline and 9-month follow-up protocols.

### Baseline characteristics

Randomization resulted in comparable intervention and control groups at baseline (see Table 1). Females numbered 71(79.8 %); 34 (81.0 %) were in the intervention group, and 37 (78.0 %) in the control group. There were no significant differences between intervention and control group on any variables at baseline.

**Table 1:** Characteristics of participants at baseline

Variable	Intervention group *n* = 42	Control group *n* = 47	*P*-value
Sex (male/female)	8/34	10/37	0.794^a^
Age in years (mean ± SD)	75.32 ± 5.67	75.81 ± 6.97	0.715^b^
Previous history of clinical symptoms			
Bone and joint disease	28	33	0.719^a^
Nucleus disease	5	2	0.173^a^
Heart trouble	5	3	0.295^a^
Hypertention	14	22	0.196^a^
Diabetes	3	5	0.422^a^
Digestive system disease	4	6	0.444^a^
Depression	1	2	0.543^a^
QOL (Quality of Life)			
Self-rated health (five methods)	3.14 ± 0.78	3.23 ± 0.84	0.599^b^

Data are mean and SD

^a^ *P*-values describe Chi-square test

^b^ *P*-values describe Student’s t-test

### Intervention-related changes in outcomes

LGST produced improvements on the frailty criteria (Table 2). The intervention group had accumulated 26 (61.9 %) of the possible improvement points at the 3-month interval, and maintained this gain at 6 months, with a slight (but statistically non-significant) additional improvement at 9 months (27 points, 64.3 %). The mean improvement over all intervals was 62 %. In contrast, the control group scored 19 (40.4 %), 17 (36.2 %) and 22(46.8 %) at the 3-, 6- and 9-month intervals, respectively, for a mean improvement of 41 % over all intervals. The group difference was significant at the 3-month (*p* = 0.043) and the 6-month (*p* = 0.015), but not at the 9-month (*p* = 0.098) interval.

**Table 2:** Values of improvement in frailty criteria for the intervention and control groups

Variable	Baseline	*P*-value	3 months	*P*-value	6 months	*P*-value	9 months	*P*-value
Improvement in the frailty criteria in the Kihon Checklist (%)								
Intervention (*n* = 42)	0	NA	26 (61.9)	0.043^*^	26 (61.9)	0.015^*^	27 (64.3)	0.098
Control (*n* = 47)	0		19 (40.4)		17 (36.2)		22 (46.8)	

*NOTE*: Values expressed as number of health improvement points

*NA* not applicable

In the QOL analysis, the intervention group scored 3.14 (SD 0.78) at baseline, 3.81(SD 0.80) at 3 months, 3.50 (SD 0.74) at 6 months and 3.36 (SD: 0.66) at 9 months. The respective control group scores were 3.23 (SD 0.84), 3.30 (SD 0.81), 3.23 (SD 0.91) and 3.23(SD 0.91). The differences yielded a significant time × group interaction effect (*p* = 0.022). This effect owed solely to improvements at 3-month interval in the intervention group; no effect was revealed in the control group at any time (Table 3).

**Table 3:** Mean values of QOL for the intervention and control groups

Variable	Baseline	3 months	*P*-value**	6 months	*P*-value**	9 months	*P*-value**	*P*-value*
QOL (Quality of Life): self-rated health (the five methods)								
Intervention (*n* = 42)	3.14 ± 0.78	3.81 ± 0.80	0.001*	3.50 ± 0.74	0.078	3.36 ± 0.66	0.415	0.022*
Control (*n* = 47)	3.23 ± 0.84	3.30 ± 0.81	0.970	3.23 ± 0.91	1.000	3.23 ± 0.91	1.000	

*NOTE*: Values expressed as number of health improvement points

*P-values describe the time × group interaction effect

**P-values describe Dunnett’s multiple comparison test

### Secondary outcomes

#### Life goal

Life goal recognition scores for the intervention group were 40 (95.2 %) at 3-month, 36 (85.7 %) at 6-month, and 41 (67.6 %) at 9-month. The 3-month scores related to life goals made with the OT at the beginning of the classroom program, and the 6- and 9-month scores related to new life goals made at the end of the classroom program. For the control group, recognition was 36 (76.6 %) at 3-month, 33 (70.2 %) at 6-month and 31 (66.0 %) at 9-month. All the control group scores related to life goals made with the care manager before the classroom program. The difference was significant at 3-month (*p* = 0.013) and 9-month (*p* = 0.001) intervals, but not at the 6-month interval (*p* = 0.051). The recognition rate was significantly higher in the intervention group than in the control group at 3 and 9 -months (Table 4).

**Table 4:** Values of degree of recognition, achievement and satisfaction of the life goal

3 months						
Variable	Recognition of the life goal (Yes)(%)	*P*-value^d^	Achievement of the life goal (the 10 methods)	*P*-value^e^	Satisfaction of the life goal (the 10 methods)	*P*-value^e^
Intervention (*n* = 42)	40(95.2)^a^	0.013*	7.00 ± 2.53^a^	0.961	8.00 ± 2.68^a^	1.000
Control (*n* = 47)	36(76.6)^c^		6.97 ± 2.36^c^		8.00 ± 2.68^c^	
6 months						
Variable	Recognition of the life goal (Yes)(%)	*P*-value^d^	Achievement of the life goal (the10 methods)	*P*-value^e^	Satisfaction of the life goal (the 10 methods)	*P*-value^e^
Intervention (*n* = 42) 36(85.7)^b^	36(85.7)^b^	0.051	7.28 ± 2.77^b^	0.105	8.00 ± 2.29^b^	0.012*
Control (*n* = 47)	32(68.1)c		6.38 ± 2.62^c^		6.19 ± 2.60^c^	
9 months						
Variable	Recognition of the life goal of the life goal (Yes)(%)	*P*-value^d^	Achievement of the life goal (the 10 methods)	*P*-value^e^	Satisfaction of the life goal (the 10 methods)	*P*-value^e^
Intervention (*n* = 42)	41(97.6)^b^	0.001**	7.49 ± 2.21^b^	0.025*	7.41 ± 2.40^b^	0.019*
Control (*n* = 47)	32(68.1)^c^		6.38 ± 2.62^c^		6.19 ± 2.60^c^	

*NOTE*: Values expressed as number (%) or mean and SD

^a^The life goals which were *made with the OT* at the beginning of the classroom program

^b^The new life goals, *revised with the OT* at the end of the classroom program

^c^The life goals which were made *with the care manager* before the classroom program

^d^ *P*-values describe Chi-square test

^e^ *P*-values describe Student’s t-test

The mean score for achievement of life goals, for those in the intervention group who recognized their life goal, was 7.00 at 3 months. Scores for achievement of the new life goal were 7.28 at 6 months, and 7.49 at 9 months. For the control group, achievement scores were 6.97 at 3 months, and 6.38 at 6 and 9 months. The difference was significant only at 9 months (*p* = 0.025). The achievement rate was significantly higher for the intervention group than the control group at 9-month (Table 4).

Scores for satisfaction of the life goal for those in the intervention group who recognized their life goals were 8.00 at 3- and 6-month, and 7.41 at 9-month. For the control group, satisfaction was 8.00 at 3-month, and 6.19 at 6- and 9-month. The difference was significant at 6-month (*p* = 0.012) and 9-month (*p* = 0.019), but not at 3-month (*p* =1.000). The satisfaction rate was significantly higher for the intervention group than for the control group at 6 and 9 months (Table 4).

#### Physical function

In the intragroup comparison between baseline and 3 months, the intervention group showed significant differences in physical functions: grip strength (*p* = 0.010), sit and reach test (*p* = 0.001), Timed Up-and-Go test (*p* = 0.027). The control group showed no significant differences on any physical functions. There was no significant time × group interaction (Table 5).

**Table 5:** Physical performance test for the intervention and control groups

Variable	Baseline	Baseline (*P* value***)	3 months	Baseline-3 months (*P*-value**)	*P*-value*
Grip strength (kg)					
Intervention (*n* = 42)	20.86 ± 5.71	0.590	21.94 ± 4384	0.010*	0.108
Control (*n* = 47)	21.53 ± 5.89		21.82 ± 5.98	0.202	
Sit and reach test (cm)					
Intervention (*n* = 42)	31.83 ± 9.59	0.245	34.38 ± 9.84	0.001^**^	0.382
Control (*n* = 47)	34.15 ± 0.89		35.75 ± 9.20	0.125	
Timed Up-and-Go Test (min)					
Intervention (*n* = 42)	8.61 ± 2.68	0.34	8.11 ± 2.85	0.027^*^	0.811
Control (*n* = 47)	9.10 ± 2.09		8.50 ± 3.13	0.077	

*NOTE*: Values expressed as mean ± SD

*P-values describe the time × group interaction effect

**P-values describe paired t-test

***P-values describe independent t-test

## Discussion

The results of this study showed that the 3-month SPCP plus LGST intervention improved health immediately after the intervention and at follow-up to 6 months. It also produced immediate QOL improvement after the intervention. For frailty criteria, gains were present at both 3 months and 6 months, but failed to reach significance at 9 months.

QOL gains were seen at 3 months, but were not significantly different at 6 or 9 months.

The intervention also accounted for significant differences between the two groups on recognition of life goals at 3 and 9 months. The difference at 6 months approached significance. Life goals were associated with health and QOL improvement.

### Effects of the 3-month program

There were no significant differences between the two groups (time × group) for the index of physical functions. However, the intervention group showed a significant difference at baseline and 3 months. Results suggest that the intervention group has more effective of physical functions. A previous systematic review has shown that exercise seems to be beneficial in improving physical function [8]. Almost of the trials reported statistically significant effects for mobility, balance, functional ability, muscle strength and body composition [30–32]. These trials intervention were 12 weeks and frequency of the training programs 2times per week for community-dwelling older in all trials.

Results suggest that the 3-month SPCP plus LGST was more effective than SPCT alone for improvement of health and QOL for frail community-dwelling older people.

The present review found that exercise training did not have a statistically significant impact on QOL in frail older adults [7, 8]. However previous studies [33–35] reported that improvements in physical function and performance in ADLs resulting from exercise might stimulate individuals to engage in activity or social participation and improve QOL. We found a similar result in our study of goal-setting among elderly care home residents [33, 36–38]. Goals setting are one of the most important approaches of human motivation [39]. The participants of The SPCP plus LGST might have more motivation and be more engaged in activity or social participation and achievement than control group. Thus, the negative answer of the Kihon checklist and the index of the frailty might be decreased, so to be healthy might be increased. About improvement health, the MHLW showed that the improvement percentage of preventive care program participants was 42.0 % in 2011 [29]. The improvement percentage of the control group in this study was similar at 40.4 %. For the intervention group, it was significantly higher at 61.9 %.

The fraction of participants who recognized a life goal was 20 % higher in the intervention group at 3 months. In both groups, those who recognized a life goal experienced nearly equal levels of achievement and satisfaction. The all participants of this study decided to participate in a care prevention program for own intention, and attended more than 8 of 12 times, it is thought that there is motivation to want to improve health. In addition, for intervention group, OTs set small steps for participants’ life goals, to be achievable during the program. These fractions of recognition of a life goal and achievable steps likely interacted with QOL.

There are three points that should be emphasized about recognizing life goals. The first is the timing of goal-setting. The intervention group developed their life goals with the OT at the beginning and end of the program. Life goals for members of the control group were made with the care manager, alongside the care plan, before the program. Therefore, at 3 months there had been a time lag after goal-setting. The second point is that the intervention group’s goal-setting included defining activities and participation levels that were tailored to the individuals, and were more concrete within the context of their personal living conditions and values. In contrast, the control group’s goals were generally about health or mind and body functional levels. Examples included “to maintain my health” or “to improve the pain in my knee.” The intervention group seemed to benefit from having goals set within the context of their personal living conditions and values. The third point relates to the strategy for recognizing life goals. The intervention group’s life goals were verbalized, but also clarified on paper (life goal-setting sheet) by the OT, and to promote their recognition and ownership by participants, the life goals and action plans were also shared with other staff. Our results imply that the specific strategies the intervention employed for goal-setting and goal reinforcement, including strategies related to activities and participation levels, may have played an important role in improving health and QOL outcomes.

### The durability of the intervention effect

The durability of improvement of health in both groups at 6 and 9 months was almost maintained. There was a significant statistical difference at 6-month, but not at 9-month. On the QOL, there was no significant statistical difference between two groups and post hoc test. We did not measure the durability of physical function improvement.

The goal setting is the use of usual activities as exercise acceptable to older people, and has potential for long-term compliance [40]. The durability of improvement of health and QOL seem to benefit from LGST, although the relation remains unclear.

The rate of recognition of a life goal was high in the intervention group, but low in the control group. There was a significant statistical difference at 9 months, and a tendency toward significance at 6 months. Recognition of life goals appears to influence the durability of the improvement ratio. The degree of achievement of the life goal and the durability of satisfaction were also different between groups at 9 months.

The intervention group set and staged a new life goal at the end of the program, matched to their ability after the program. In addition the intervention group’s new life goals were also clarified on paper at the end of the program. There was therefore a difference in the number of times that the groups looked at life goals. Recognition in the control group decreased over time, but was maintained for the intervention group. We used a postal questionnaire to confirm the percentage of recognition of goals for both groups at 6 and 9 months after the end of the classroom program. The members of both groups may therefore have reviewed the goals again after the program or on receipt of the postal questionnaire, which could have helped with recognition. At the end of the program, they therefore reviewed their lives and made changes to what they wanted to do. Those goals were usually matched participants’ abilities after the program.

### Strengths and weaknesses of the study

The strengths of this cluster randomized trial include a long follow-up period and similarity between the groups at baseline. Also, the preventive methods were multifactorial, individually tailored and based on exercise, oral care and nutrition education and support for achieving life goals within the individual’s lifestyle. The sample size was estimated using power calculations.

For frail older people, it has been reported previously that individual diversity assessments and intervention models are necessary. Goal-setting was included in the Dutch EASY care study [41] and general practice projects [33, 36–38], but the main intervention was home visits.

This study also has some weaknesses. Significantly more participants were lost to follow-up in the intervention group than in the control group (47 v 25 %). A previous review [7] showed the dropout rate in five of the eight trials did not exceed 15 %; yet, this study’s dropout rate was larger. We cannot fully explain this finding, because participants were excluded if they missed the interview with the OT either at the beginning or end of the program. Second, our sample was 80 % female. Females were oversampled to avoid contamination bias. Previous reviews of community-dwelling adults have also had high percentages (60–70 %) of females [8, 30, 31, 38, 42]. Previous reports have also found that social participation produces bigger benefits for the health of women than for men [43, 44], so the reported rates in our study may overestimate improvements for men.

### Limitations

The design of this cluster nonrandomized controlled trial resulted in a few problems in estimating required group size and number. We used slightly different outcome measures from previous studies, which made it harder to compare our results with others. For example, for the primary outcome of frailty, we used the Kihon Checklist. Other studies have used the Comprehensive Geriatric Assessment (CGA) [4, 6, 14] and the Groningen Activity Restriction Scale [38–40]. For QOL, we used a 5-item measure of self-rated health, unlike previous studies, which used the Medical Outcomes Study (MOS-20) [38] and the Older People’s Quality Of Life (OPQOL) questionnaire [4].

We suggest that a larger study over a wider geographic area is required in future.

We did not follow up on oral health, torpidity or motor function, so the long-term effect on these elements is unclear. We were also unable to establish the mechanism for influencing activity and participation. Future studies will need to look at these influences and association durability of interventions with oral health, overall physical activity and motor function.

## Conclusions

A 3-month exercise program helped to decrease frailty and improve QOL in frail older people, and the addition of LGST increased its effectiveness. For frail older people, we determined that recognition of a life goal is important. We suggest that the specific strategies the intervention employed for goal-setting and goal reinforcement, including strategies related to activities and participation levels, may have played an important role in improving health and QOL outcomes. The LGST is a feasible and promising intervention for reducing risk of needing care. To improve health and QOL, further research should add the LGST in a follow-up period of preventive care program in frail older people.

## Availability of supporting data

Data files are not available due to participants’ confidentiality.

## Abbreviations

ADL: activities of daily living
CGA: comprehensive geriatric assessment
COPM: Canadian Occupational Performance Measure
IADL: instrumental activities of daily living
KCL: Kihon checklist
LGST: life goal-setting technique
MHLW: Ministry of Health, Labour and Welfare
MOS: Medical Outcomes Study
OPQOL: older people’s quality of life questionnaire
OT: occupational therapist
PHN: public health nurse
QOL: quality of life
SPCP: standard preventive care program
