# Erratum to: The effects of a life goal-setting technique in a preventive care program for frail community-dwelling older people: a cluster nonrandomized controlled trial

**DOI:** 10.1186/s12877-017-0488-2

**Published:** 2017-05-08

**Authors:** Yoshimi Yuri, Shinichi Takabatake, Tomoko Nishikawa, Mari Oka, Taro Fujiwara

**Affiliations:** 1 Department of Rehabilitation Sciences, Faculty of Allied Health Sciences, Kansai University of Welfare Sciences, Osaka, Japan; 2 Graduate School of Comprehensive Rehabilitation, Osaka Prefecture University, Osaka, Japan; 3 Elderly Care Office, Izumi, Osaka, Japan; 4 Izumi Rehabilitation Home-Visit Nursing Care Station, Osaka, Japan

## Erratum

After the publication of this work [1] an error was discovered in the calculation of the sample size. This error does not substantially alter the results or conclusions. The following paragraph in the section 'Sample size and Power' has been modified as follows:

**Original text.**

Based on a previous report [29], we expected to demonstrate a difference of at least 7% in mean change in score of the frailty checklist between the intervention and control groups (equivalent to an effect size of 0.43). Based on a power of 80% and an alpha of 0.05, we decided on a minimum sample size of *n* = 32 per group (64 in total). Based on expected drop-out rate of 50% in the control group and 60% in the intervention group, the required sample size was *n* = 64 for the control group and *n* = 80 for the intervention group (*n* = 144 in total).

**Revised text.**

Based on a previous report [29], we expected to demonstrate a difference of at least 20% in mean change in score of the frailty checklist between the intervention and control groups (equivalent to an effect size of 0.41). Based on a power of 80% and an alpha of 0.05, we decided on a minimum sample size of *n* = 47 per group (94 in total). Based on an expected drop-out rate of 30% in the control group and 40% in the intervention group, the required sample size was *n* = 67 for the control group and *n* = 78 for the intervention group (*n* = 145 in total).

It was also discovered after publication that there was a numerical error in the 3rd row, 4th column of table 5. The entry “21.94 ± 4384” should be “21.94 ± 4.84”.
